# The neutrophil–osteogenic cell axis promotes bone destruction in periodontitis

**DOI:** 10.1038/s41368-023-00275-8

**Published:** 2024-02-27

**Authors:** Yutaro Ando, Masayuki Tsukasaki, Nam Cong-Nhat Huynh, Shizao Zang, Minglu Yan, Ryunosuke Muro, Kazutaka Nakamura, Masatsugu Komagamine, Noriko Komatsu, Kazuo Okamoto, Kenta Nakano, Tadashi Okamura, Akira Yamaguchi, Kazuyuki Ishihara, Hiroshi Takayanagi

**Affiliations:** 1 Department of Immunology, Graduate School of Medicine and Faculty of Medicine, The University of Tokyo, 7-3-1, Hongo, Bunkyo-ku, Tokyo Japan; 2 Department of Microbiology, Tokyo Dental College, 2-1-14 Kanda-Misaki-cho, Chiyoda-ku, Tokyo Japan; 3 Oral Health Science Center, Tokyo Dental College, 2-9-18, Kanda-Misaki-cho, Chiyoda-ku, Tokyo Japan; 4 Department of Osteoimmunology, Graduate School of Medicine and Faculty of Medicine, The University of Tokyo, 7-3-1, Hongo, Bunkyo-ku, Tokyo Japan; 5 Unit of Prosthodontics, Laboratory of Oral-Maxillofacial Biology Faculty of Odonto-Stomatology, University of Medicine and Pharmacy at Ho Chi Minh City, Ho Chi Minh City, Vietnam; 6 Department of Oral and Maxillofacial Surgery, Department of Sensory and Motor System Medicine, Graduate School of Medicine, The University of Tokyo, Tokyo, Japan; 7 Division of Rheumatology, Department of Internal Medicine, Keio University School of Medicine, Tokyo, Japan; 8 Department of Laboratory Animal Medicine, Research Institute, National Center for Global Health and Medicine, Tokyo, Japan

**Keywords:** Periodontitis, Bone

## Abstract

The immune-stromal cell interactions play a key role in health and diseases. In periodontitis, the most prevalent infectious disease in humans, immune cells accumulate in the oral mucosa and promote bone destruction by inducing receptor activator of nuclear factor-κB ligand (RANKL) expression in osteogenic cells such as osteoblasts and periodontal ligament cells. However, the detailed mechanism underlying immune–bone cell interactions in periodontitis is not fully understood. Here, we performed single-cell RNA-sequencing analysis on mouse periodontal lesions and showed that neutrophil–osteogenic cell crosstalk is involved in periodontitis-induced bone loss. The periodontal lesions displayed marked infiltration of neutrophils, and in silico analyses suggested that the neutrophils interacted with osteogenic cells through cytokine production. Among the cytokines expressed in the periodontal neutrophils, oncostatin M (OSM) potently induced RANKL expression in the primary osteoblasts, and deletion of the OSM receptor in osteogenic cells significantly ameliorated periodontitis-induced bone loss. Epigenomic data analyses identified the OSM-regulated RANKL enhancer region in osteogenic cells, and mice lacking this enhancer showed decreased periodontal bone loss while maintaining physiological bone metabolism. These findings shed light on the role of neutrophils in bone regulation during bacterial infection, highlighting the novel mechanism underlying osteoimmune crosstalk.

## Introduction

The oral cavity, a gateway to the digestive and respiratory systems, harbors an estimated 1 000 bacterial species.^1^ Dysbiosis in oral microbial communities stimulates host immune responses and causes periodontitis, the most prevalent infectious disease in humans.^2–5^ Periodontitis is the primary cause of adult tooth loss and also affects various systemic diseases including diabetes, pneumonia, cardiovascular diseases, rheumatoid arthritis (RA), and inflammatory bowel disease.^6–11^ Thus, a detailed understanding of the cellular and molecular mechanisms underlying oral mucosal immunopathology is critical for developing therapeutic and preventive strategies against periodontitis and its comorbidities.

Recent advances in single-cell and spatial multiomics technologies have provided a cellular landscape of oral mucosal lesions, critically contributing to our understanding of the pathogenesis underlying periodontitis.^12–17^ Previous studies have shown that various immune cells, including T cells, B cells, and myeloid cells, accumulate in periodontitis lesions and may interact with stromal cells such as fibroblasts and epithelial cells.^12,15–18^ However, the in vivo relevance of immune–stromal cell interactions in periodontitis has not been proven by loss-of-function approaches, and it remains unclear how the complex cellular interactions ultimately induce osteoclastic bone resorption.

Experimental animal models are critical tools to investigate the mechanisms of disease pathogenesis. The ligature-induced periodontitis mouse model is the most widely used experimental model in the field, and genetic loss-of-function studies using this model have revealed that various immune cells such as T helper 17 (T_H_17) cells play an essential role in periodontal bone destruction.^4,19,20^ Receptor activator of nuclear factor-κB ligand (RANKL), the master cytokine inducing osteoclast differentiation and activation, is mainly produced by osteogenic cells including osteoblasts and periodontal ligament (PDL) cells during periodontitis.^4,21,22^ Inflammatory cytokines such as interleukin 17 A/F (IL-17A/F) and IL-6 stimulate RANKL expression in osteoblasts and PDL cells.^4^ However, the genetic deletion of these cytokines only partly inhibits periodontitis-induced bone destruction, suggesting that other factors may also contribute to the induction of RANKL in osteogenic cells.^4,22^ To obtain a comprehensive picture of the pathogenesis of periodontitis, it is necessary to understand the detailed molecular mechanisms underlying RANKL induction in osteogenic cells during periodontal inflammation.

In this study, we demonstrated that the neutrophil–osteogenic cell interaction promotes bone destruction in periodontitis through oncostatin M (OSM)/OSM receptor (OSMR) signaling. Single-cell RNA sequencing (scRNA-seq) analyses of the mouse periodontal lesions revealed that inflammatory neutrophils markedly accumulated in the periodontal lesions. Inflammatory neutrophils highly expressed OSM, which strongly induced RANKL expression in osteogenic cells. Periodontitis-induced bone loss was inhibited in osteogenic cell-specific OSMR-deficient mice, demonstrating the in vivo relevance of OSM-mediated neutrophil–osteogenic cell crosstalk. We identified the OSM-regulated RANKL enhancer region in osteogenic cells, and mice lacking this enhancer displayed decreased periodontal bone loss while maintaining physiological bone metabolism. Together, the results of this study reveal the detailed mechanisms underlying bone damage associated with periodontitis, providing a novel example of immune–stromal cell interactions in infectious diseases.

## Results

### Single-cell landscape of mouse periodontal lesions

**Fig. 1:**
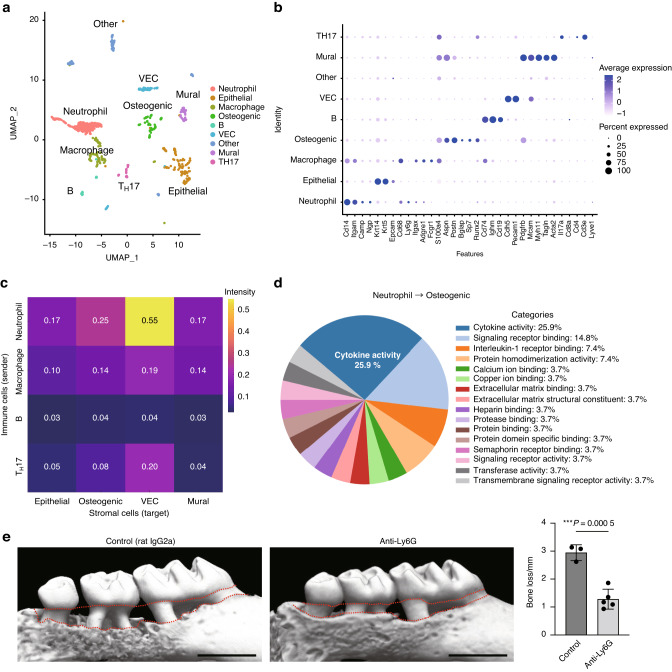
Neutrophils interact with osteogenic cells through cytokine production in periodontitis. **a** Uniform manifold approximation and projection (UMAP) plot of cells from the periodontal tissues of ligature-induced periodontitis mice (*n* = 5). **b** Dot plot of the expression of representative genes for each cluster. Each cell type was annotated using these marker genes. **c** Heat map showing the network intensity of interactions between immune cells (sender) and stromal cells (target). **d** Proportion of Gene Ontology terms on molecules mediating the effect of neutrophils on osteogenic cells. **e** Micro-CT analysis of periodontitis-induced bone loss in mice treated with isotype-control (rat IgG2a) and anti-Ly6G antibodies (*n* = 3 and *n* = 5, respectively). Scale bars, 1 mm. The upper red dotted line indicates the cement–enamel junction, and the lower red dotted line indicates the alveolar bone crest in the left panel. The periodontal bone loss was quantified in the right panel

To understand the cellular microenvironment underlying periodontal bone destruction at a single-cell resolution, we performed scRNA-seq using cells derived from the periodontal tissue of ligature-induced periodontitis mice at 7 days after ligature placement when the marked infiltration of immune cells and accelerated bone destruction were observed in previous studies.^4,19,20,23^ Unsupervised graph-based clustering analysis showed that the mouse periodontal lesions comprised immune cells (neutrophils, macrophages, T_H_17 cells, and B cells), epithelial cells, vascular endothelial cells (VEC), mural cells, and osteogenic cells (Fig. 1a, b). We annotated the osteogenic cell cluster, which is characterized by the expression of marker genes for both osteoblasts (*Sp7* and *Runx2*) and PDL cells (*S100a4* and *Postn*). Since PDL cells have osteogenic capacity and serve as the local source of osteoblasts in vivo, it is difficult to rigorously discriminate between PDL cells and osteoblastic cells using sequencing data.^24,25^

To gain insights into the role of immune–stromal cell interactions in bone destruction associated with periodontitis, we performed CellChat analysis, a quantitative tool for measuring intercellular signaling networks, using our scRNA-seq data.^26^ We calculated the mean interaction intensity between immune cells and stromal cells, and found that the interplay between neutrophils and osteogenic cells was pronounced among intercellular interactions in the periodontal lesions (Fig. 1c, Supplementary Fig. S1a). The interaction between neutrophils and VEC in the periodontitis pathogenesis has been well documented by previous studies,^27–30^ but the role of neutrophil-osteogenic crosstalk in periodontitis has never been reported. Since osteogenic cells represent the major source of RANKL in the periodontal bone damage, we focused on the neutrophil-osteogenic axis. The CellChat analysis data suggested that osteogenic cells act on neutrophils by expressing various factors including cell adhesion molecules and chemokines (Supplementary Fig. S1b). The effect of neutrophils on osteogenic cells is suggested to be largely dependent on the cytokines produced by neutrophils (Fig. 1d). To assess the importance of neutrophils in the periodontal bone destruction in vivo, we depleted neutrophils by injecting anti-Ly6G antibody, which led to the significant inhibition of periodontitis-induced bone loss (Fig. 1e). These findings suggest that neutrophils may interact with osteogenic cells through cytokine production and promote bone destruction in periodontitis.

### OSM stimulates RANKL expression in osteogenic cells

**Fig. 2:**
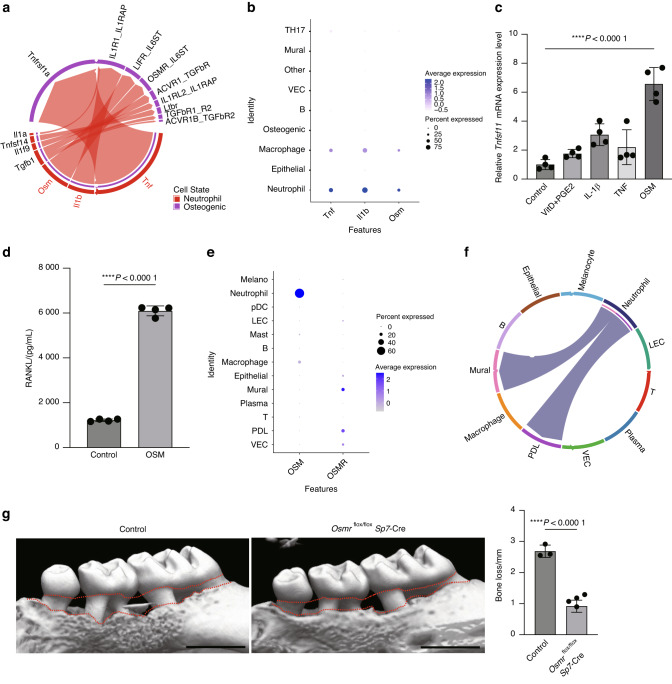
OSM stimulates RANKL expression in osteogenic cells and promotes bone destruction in periodontitis. **a** Chord diagram showing cytokine signaling interaction pairs in the neutrophil-osteogenic network. **b** Dot plot showing the expression of TNF, IL-1β, and OSM in the scRNA-seq clusters of the mouse periodontitis lesion. **c** qPCR analysis of *Tnfsf11* transcripts in calvaria-derived primary osteoblasts treated with TNF, IL-1β, OSM or Vitamin D plus Prostaglandin E2 (PGE2) (*n* = 4). The data were obtained from duplicate experiments. **d** RANKL concentration measured by ELISA in the lysate of primary osteoblasts treated with or without OSM. **e** Dot plot showing the expression of OSM and OSMR in scRNA-seq clusters of the human periodontitis lesion. **f** Chord diagram showing the OSM signaling interaction pairs in the cell-cell network of the human periodontitis lesion. **g** Micro-CT analysis of periodontitis-induced bone loss in the control (*n* = 3) and *Osmr*^flox/flox^ *Sp7*-Cre mice (*n* = 5). Scale bars, 1 mm. The upper red dotted line indicates the cement–enamel junction and the lower red dotted line indicates the alveolar bone crest in the left panel. The periodontal bone loss was quantified in the right panel

Since osteogenic cells function as the primary source of RANKL in periodontal bone loss, we hypothesized that neutrophils may contribute to periodontal bone loss by inducing RANKL expression in osteogenic cells through cytokine production. We analyzed the CellChat data by focusing on the cytokine-mediated interaction between neutrophils and osteogenic cells, and found that tumor necrosis factor (TNF), IL-1β, and OSM displayed high communication intensity in the neutrophil-osteogenic network (Fig. 2a). These cytokines are specifically expressed in neutrophils in the periodontal lesion (Fig. 2b). Among the three cytokines, OSM had the strongest capacity to induce RANKL expression in mouse calvaria-derived primary osteoblasts in vitro (Fig. 2c). We confirmed that OSM significantly upregulates the expression of osteoblastic RANKL at the protein level (Fig. 2d). The scRNA-seq data analysis of human periodontal lesions also showed that OSM and OSMR were highly expressed in neutrophils and PDL cells, respectively (Fig. 2e). The CellChat analysis suggested that the OSM/OSMR axis also mediates the neutrophil-PDL interaction in human periodontal lesions (Fig. 2f).

To investigate the importance of OSMR signaling in osteogenic cells in periodontitis-induced bone damage in vivo, we crossed OSMR-floxed mice with *Sp7*-Cre mice. In the periodontal tissue, *Sp7*-Cre was shown to target both osteoblasts and PDL cells.^22^ Because OSMR signaling in osteoblastic cells is involved in physiological bone metabolism,^31^ we used a *Sp7*-tTA-tetO-Cre (*Sp7*-Cre) system in which Cre recombinase is expressed only when a tetracycline-controlled transactivator (tTA) binds to a tetracycline responsive element (tetO) in the absence of doxycycline (Dox). *Sp7*-Cre mice crossed with OSMR-floxed mice were treated from the prenatal period with Dox, which was withdrawn at the age of 4 weeks. This protocol did not affect skeletal growth in the mice (Supplementary Fig. S2a–c). We induced periodontitis at the age of 8 weeks, and the alveolar bone was analyzed 10 days after ligature placement. Notably, periodontitis-induced bone loss was significantly inhibited in the *Osmr*^flox/flox^ *Sp7*-Cre mice (Fig. 2g). These results suggest that neutrophil-derived OSM binds to the OSMR on osteogenic cells and promotes RANKL expression, underscoring the relevance of the neutrophil–osteogenic cell interaction in bone destruction associated with periodontitis.

### Identification of the OSM-regulated RANKL enhancer region

**Fig. 3:**
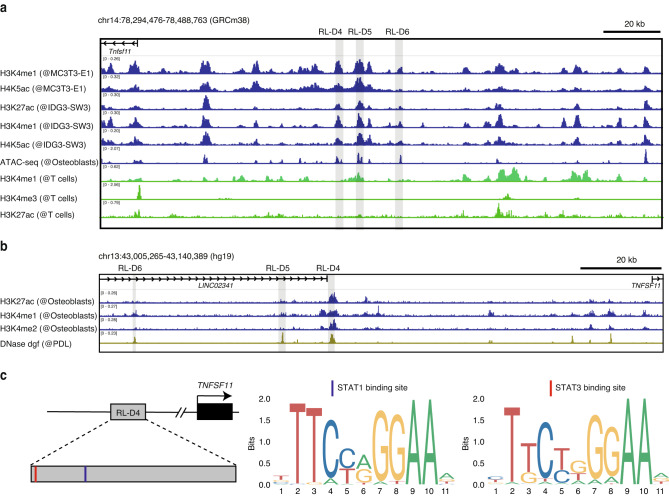
Identification of the OSM-regulated RANKL enhancer region in osteogenic cells. **a** H3K4me1, H3K4me3, H4K5ac, and H3K27ac ChIP-seq and ATAC-seq profiles in mouse osteogenic cells (GSE51515, GSE54782, and GSE174045) and T cells (ENCFF021TWR). The gray shaded areas indicate the RANKL distal enhancer regions (RL-D4, RL-D5, and RL-D6). **b** H3K27ac, H3K4me1, and H3K4me2 ChIP-seq profiles in human osteogenic cells (GSE29611) and DNase-seq profile in human PDL fibroblasts (ENCFF021TWR). The gray shaded areas indicate the human homologous areas of the RL-D4, RL-D5, and RL-D6 regions. **c** Schematic depicting the STAT-binding motifs and the predicted binding sites within the human homologous region of RL-D4

OSM stimulates RANKL expression in osteoblastic cells by activating the signal transducer and activator of transcription 3 (STAT3) transcription factor. In addition, three STAT3-binding sites upstream of the *Tnfsf11* locus, namely RANKL distal enhancer 4 (RL-D4), RL-D5, and RL-D6, have been reported (Fig. 3a).^32,33^ We analyzed various publicly available epigenomic datasets of mouse osteoblastic cells and found that active enhancer markers including histone H3 lysine 27 acetylation (H3K27ac), histone H3 lysine 4 monomethylation (H3K4me1), and histone H4 lysine 5 acetylation (H4K5ac) as well as assay for transposase-accessible chromatin with sequencing (ATAC-seq) peak were enriched in these STAT3-binding sites, suggesting that these regions may function as active enhancers in osteoblastic cells.

In human datasets, the homologous region of RL-D4 displayed the highest enrichment of active enhancer markers in osteoblastic cells and PDL cells (Fig. 3b). We confirmed that the binding motifs of STAT transcription factors were present in the human homologous region of RL-D4 (Fig. 3c). Previous studies have shown that deletion of RL-D5 or RL-D6 partly inhibits OSM-induced RANKL expression in osteoblastic cells, but the importance of RL-D4 in physiological or pathological bone metabolism has not been explored.^32–35^ Therefore, we evaluated the role of the RL-D4 region in periodontitis-induced bone loss by generating mice lacking the RL-D4 region using clustered regularly interspaced palindromic repeats (CRISPR)/CRISPR-associated protein 9 (Cas9)-mediated genome editing technology. The successful generation of RL-D4-knockout (KO) mice was confirmed by Sanger DNA sequencing (Supplementary Fig. S3a). The RL-D4-KO mice were born at the expected Mendelian frequency and displayed normal tooth eruption and a body size similar to that of littermate wild-type (WT) controls (Supplementary Fig. S3b).

### Periodontitis-induced bone loss is inhibited in RL-D4-deficient mice

**Fig. 4:**
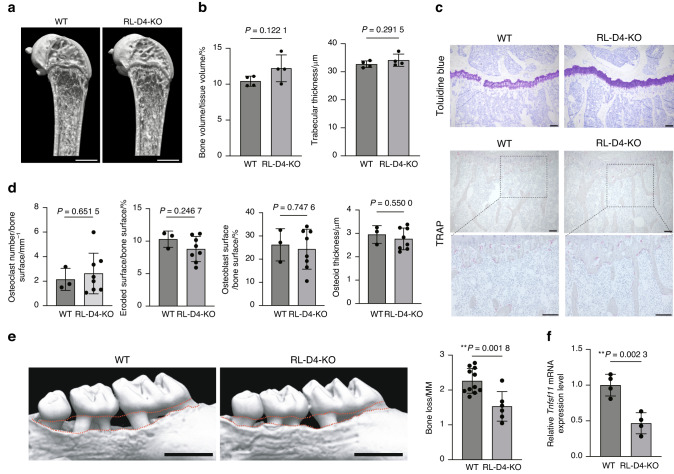
The RANKL enhancer RL-D4 is involved in periodontitis-induced bone loss but not in physiological bone remodeling. Representative micro-CT images (**a**) and micro-CT parameters (**b**) of the femur in female WT and RL-D4-KO mice at the age of 12 weeks (*n* = 4 and *n* = 4). Scale bars, 1 mm. **c** Toluidine blue and TRAP staining of the proximal tibias of WT and RL-D4-KO mice at the age of 12 weeks. The data are representative of at least three independent experiments. Scale bars, 100 μm. **d** Bone histomorphometric analysis of the proximal tibias of WT and RL-D4-KO mice at the age of 12 weeks (*n* = 3 and *n* = 8). **e** Micro-CT analysis of periodontitis-induced bone loss in WT (*n* = 11) and RL-D4-KO mice (*n* = 5). The upper red dotted line indicates the cement–enamel junction and the lower red dotted line indicates the alveolar bone crest in the left panel. Scale bars, 1 mm. Periodontal bone loss was quantified in the right panel. **f** qPCR analysis of the *Tnfsf11* transcripts in the calvaria-derived primary osteoblasts from WT mice and RL-D4-KO mice treated with OSM (*n* = 4). The data were obtained from duplicate experiments

To investigate the importance of the RL-D4 region in physiological bone remodeling, we first analyzed the long bones of RL-D4-KO mice at steady state. Microcomputed tomography (micro-CT) analyses showed that RL-D4-KO mice had normal bone phenotype compared to WT mice under physiological conditions (Fig. 4a, b). Dynamic bone histomorphometry showed no difference between WT and RL-D4-KO mice in both osteoclastic and osteoblastic parameters (Fig. 4c, d).

To assess the involvement of RL-D4 in periodontal bone damage, we induced experimental periodontitis in the RL-D4-deficient mice and found that the periodontitis-induced bone loss was significantly inhibited in these animals (Fig. 4e). We isolated primary osteoblasts from the calvaria of RL-D4-deficient mice, and found that OSM-induced RANKL expression was significantly inhibited in the absence of the RL-D4 region (Fig. 4f). These data suggest that the RL-D4 region is not required for physiological bone remodeling but contributes to periodontal bone destruction, possibly by mediating OSM-induced RANKL expression in osteogenic cells.

Taken together, our findings indicate that neutrophils contribute to bone destruction in periodontitis by activating the OSMR/RL-D4/RANKL axis in osteogenic cells.

## Discussion

Neutrophils are the most abundant phagocytes in the bloodstream of humans, functioning as a key component of the innate immune system.^30,36^ Excessive neutrophil infiltration exacerbates the pathogenesis of periodontitis in humans and mice.^37,38^ Activated neutrophils promote periodontal tissue degradation by producing matrix metalloproteinases and reactive oxygen species. Recent studies have suggested that neutrophil extracellular traps may also contribute to periodontal bone destruction by activating T_H_17 cells.^39,40^ In this study, we demonstrated that neutrophils may stimulate osteogenic RANKL expression through cytokine secretion, providing novel insights into the role of neutrophils in the pathogenesis of periodontitis. In addition to OSM, our scRNA-seq data showed that periodontal neutrophils highly expressed IL-1 and TNF, suggesting that neutrophils may act as an important source of osteoclastogenic cytokines in periodontal bone loss.^41,42^ Since the marked accumulation of neutrophils was also reported in an apical periodontitis model,^43^ the neutrophil-osteogenic cell axis may have a role in bone damage associated with endodontic infection. On the other hand, neutrophils can exert anti-inflammatory and bone-protective functions as evidenced by the fact that the neutrophil depletion delayed bone regeneration in a cranial defect model.^44^ The enhanced immune imbalance and bone destruction were reported in Leukocyte Adhesion Deficiency Type I (LAD1) patients in which poor or no recruitment of neutrophils triggers overactivation of T_H_17 cells in the periodontal tissues.^45,46^ These studies showed that either too few or too many neutrophils can exacerbate periodontitis, highlighting the importance of the precise coordination of neutrophil recruitment in periodontal tissue homeostasis. Additional studies are needed to provide a comprehensive picture of the role of neutrophils in the pathogenesis of periodontitis.

OSM is involved in various inflammatory diseases such as dermatitis, RA, and colitis.^47^ The cellular source of OSM in dermatitis and RA is T cells and macrophages,^48,49^ respectively. OSM promotes osteoclastic bone resorption by increasing RANKL expression in osteogenic cells. Previous studies showed that macrophage-derived OSM promotes osteogenic differentiation of bone marrow mesenchymal stromal cells,^50,51^ and OSM could inhibit sclerostin expression in osteocytes.^31^ Thus, the cellular source of OSM is context-specific and the effect of OSM on bone is dependent on the target cell type. In both human and mouse scRNA-seq datasets, the expression levels of OSM are higher in neutrophils than in macrophages (Fig. 2b, e). Analogous to our findings in periodontitis, OSM is mainly produced by neutrophils and contributes to the pathogenesis of colitis by mediating neutrophil–stromal cell interactions, suggesting the shared mechanisms underlying mucosal immunopathologies.^52,53^ Clinically, protein levels of OSM in the gingival crevicular fluid are reportedly higher in patients with periodontitis than in healthy controls, suggesting the involvement of OSM in the pathogenesis of human periodontitis.^54^ As inhibition of the IL-23/IL-17 axis by ustekinumab is effective for the treatment of patients with severe hereditary periodontitis, inhibition of the OSM/RANKL axis may represent a potential therapeutic target to mitigate bone destruction in patients with severe hereditary periodontitis.^46^

RANKL is a multifunctional cytokine that plays an essential role in various biological systems including the bone and immune system.^5,55^ Our group and others have identified RANKL enhancer regions that function in cell type- and context- dependent manners.^32–34,56–59^ In physiological bone remodeling, the intronic enhancer, RL-D2, and RL-D5 regions control RANKL expression in mesenchymal cells including osteocytes and osteoblasts.^33,58,60^ The intronic enhancer in osteocytic cells is activated by cellular senescence signals,^58^ which were also shown to stimulate RANKL expression in periodontal ligament cells and cementoblasts.^61^ We previously demonstrated that periodontitis-induced bone loss and osteoclast development were markedly suppressed when RANKL was deleted in osteoblastic cells and periodontal ligament cells.^4^ In our scRNA-seq dataset, the osteoblastic cells (characterized by expression of *Sp7* and *Runx2*) and periodontal ligament fibroblasts (characterized by expression *S100a4* and *Postn*) were clustered as a single population due to the limited number of cells compared to previous scRNA-seq studies.^17,62^ This is a major limitation of the current study and it will be needed to clarify relative contribution of RANKL on periodontal ligament fibroblasts and osteoblasts to periodontal bone loss in future studies.

In bone destruction associated with RA, the distal enhancer E3 region regulates RANKL expression in synovial fibroblasts.^59^ The findings of the current study indicate that the STAT3-binding RL-D4 region may be involved in OSM-induced RANKL expression in osteogenic cells and contribute to bone damage in periodontitis. RL-D4 is the same region as E2 in our previous study on RA synovial fibroblasts^59^; this region did not contribute to RANKL induction in the synovial fibroblasts. These findings indicate that the osteogenic cells and synovial fibroblasts utilize distinct enhancers for RANKL regulation under inflammatory conditions. Because other members of the IL-6 family of cytokines also activate STAT3 and promote RANKL expression in osteogenic cells, it is possible that the inhibition of periodontal bone loss in RL-D4-KO mice is a combined effect of OSM and other cytokines such as IL-6. As both RL-D5 and RL-D6 regions are reportedly involved in OSM-induced RANKL expression in osteoblastic cells in vitro,^60^ further studies are required to examine the contribution of these enhancers to periodontitis-induced bone loss, and to elucidate the role of RL-D4 region beyond periodontitis.

Intriguingly, the inflammation-associated enhancers including RL-D4, RL-D5, RL-D6, and E3 are located in the intergenic region between RANKL and A-kinase anchoring protein 11, an area that has been expanded during vertebrate evolution.^63^ We suspect that the emergence of such inflammation-associated RANKL enhancers during evolution may have linked immune activation to osteoclastic bone resorption and thus driven the emergence of inflammatory bone disease, the earliest evidence of which is periodontitis-induced bone damage in a 275 million-year-old terrestrial reptile.

Recent advances in single-cell technologies have revealed cellular heterogeneity at an unprecedented level of resolution, and increasing attention has been paid to the interaction between immune and stromal cells based on the computational inference of intercellular interactions using single-cell sequencing data.^64,65^ In this study, we demonstrated that neutrophil–osteogenic cell crosstalk through the OSM/OSMR axis promotes bone damage associated with periodontitis, providing a novel example of immune–stromal interactions. Further investigations into such cellular interactions will help provide an understanding of the pathogenesis of various diseases including periodontitis.

## Methods

### Mice

All animals were maintained under specific pathogen-free conditions, and all experiments were performed with approval of the institutional review board at The University of Tokyo (Tokyo, Japan). C57BL/6 mice were purchased from CLEA Japan, Inc. (Shizuoka, Japan). *Osmr*^flox/flox^ mice were obtained from the Jaxon Laboratory. *Sp7*-Cre mice were previously described.^4,55^ RL-D4-KO mice were generated using CRISPR/Cas9-mediated genome editing technology on the C57BL/6J background. Single guide RNA targeting the sequences of the RANKL distal enhancer region (5′-TGTCCTAAAATGTTGCCATG-3′ and 5′-AATGTGAATGATCACAAGCA-3′) and hCas9 mRNA were prepared as previously described.^59^ Primers for detection of the RL-D4-KO allele were as follows: forward, 5′-TTTCGTTGCAAAGTGGGATGAAG-3′ and reverse, 5′- ACCATTAGCATTCTGGACTGAGAA-3′. The PCR product of the RL-D4-KO allele (362 base pairs) was further sequenced using the forward primer to confirm that the founder progenies harbored the enhancer region deletion (3 412 base pair deletion corresponding to GRCm39 chr14: 78,613,540–78,616,951/GRCm38 chr14: 78,376,100–78,379,511). Founder progenies were mated with C57BL/6J WT mice to generate heterozygous offspring and subsequently intercrossed to generate WT and RL-D4-KO littermate progeny. Age- and sex-matched mice were used for all of the experiments unless otherwise noted.

### Ligature-induced periodontitis mouse model

To evaluate the periodontitis-induced bone loss, a 5-0 silk ligature was tied around the maxillary left second molar, and the contralateral tooth was left unligated to serve as the baseline control, as previously described.^4,19^ The mice were sacrificed and analyzed with micro-CT 10 days after ligature application. Prior to micro-CT inspection, the maxillae were preserved in a 70% ethanol solution. Micro-CT scanning was performed with the ScanXmate-A100S Scanner (Comscantechno Co. Ltd., Kanagawa, Japan). Three-dimensional microstructural image data were reconstructed and structural indices were calculated using TRI/3D-BON bone analysis software (RATOC System Engineering Co. Ltd., Tokyo, Japan). Mice in which the ligatures were lost were excluded from the data.

### Single-cell RNA-seq and data analysis

To isolate single cells from the periodontal tissues, we collected cells from gingival tissues and the alveolar bone surface by curetting with forceps. Subsequently, the obtained periodontal tissues were cut into small pieces and digested in 2 mg/mL Collagenase Type 2 (Worthington Biochemical Corp.) and 1 mg/mL DNase Type 1 (Sigma) for 20 min at 37 °C. 0.5 mol/L EDTA was added and the tissue was incubated for another 10 min at 37 °C. After enzymatic digestion, periodontal cells were collected by centrifugation, following filtration through a 70 µm mesh. Single-cell RNA-seq analysis was performed with the 10x Genomics Chromium system (10x Genomics, Pleasanton, CA, USA). Single cells were isolated from the periodontal tissues of five mice with periodontitis. The sequencing libraries were generated from these cells using the 10x Genomics Single-cell 3′ Solution Kit (v.3) and then subjected to Illumina sequencing (HiSeq 4000 Sequencing System; Illumina, San Diego, CA, USA). Alignment and quantitation of sample count matrices were performed using the 10x Genomics Cell Ranger Pipeline (v.3.0) and mouse reference sequences (version mm10) as indicated in the manufacturer’s protocol. Downstream analysis was performed using the Seurat R package (v.4.3.0.1). We primarily filtered out genes expressed in <3 cells and cells with <200 genes. Cells with greater than 10% mitochondrial reads, 7 500 nFeature_RNA, and 60 000 nCounts RNA were also excluded. After quality control, data normalization was performed using the NormalizeData function in Seurat, and the top 2 000 variable features were identified. The Uniform Manifold Approximation and Projection dimensional reduction technique was applied to visualize the clusters at a resolution of 0.1. The cell–cell communication was analyzed using the CellChat v1.6.1R package.^26^

### Administration of anti-Ly6G antibody in vivo

Mice were administered an intraperitoneal dose of 0.4 mg of anti-Ly6G antibody (clone 1A8, BioXCell) or a rat IgG2a isotype control (clone 2A3, BioXCell). The antibody was injected on a schedule of four time points: one day before ligature placement, and 2, 5, and 8 days after ligature placement. Bone loss was subsequently measured 10 days after ligature placement.

### Cytokine stimulation assay

We isolated primary osteoblasts from the calvaria of newborns by enzymatic digestion using Alpha Minimum Essential Medium (11900024; Gibco, Waltham, MA, USA) supplemented with 0.1% collagenase (038-22361; Wako Chemicals, Osaka, Japan) and 0.2% Dispase II (383-02281; Wako Chemicals), as previously described.^58^ After digestion, osteoblasts were collected and seeded in 24-well plates. After 1day of incubation, cells were stimulated with IL-1β (R&D, 401-ML), TNF-α (R&D, 410-MT), and OSM (R&D, 495-MO) at a concentration of 50 ng/mL. After 72 h, stimulated osteoblasts were collected and subjected to quantitative PCR (qPCR) assays. In the experiment to examine the induction of RANKL expression by OSM in calvaria-derived osteoblasts from RL-D4-deficient mice, cells were collected in the same manner described above. These osteoblasts were seeded in 24-well plates. After 1 day of incubation, cells were stimulated with OSM at a concentration of 100 ng/mL. After 6 h, stimulated osteoblasts were collected and subjected to qPCR assays.

### ELISA

The isolation of the primary osteoblasts was described above. After enzymatic digestion, osteoblasts were collected and seeded in 6-well plates. After 1 day of incubation, cells were stimulated with OSM (R&D, 495-MO) at a concentration of 50 ng/mL. After 72 h, cell lysates were collected from the osteoblasts with RIPA Buffer (nacalai tesque) containing 1% Protease Inhibitor Cocktail (nacalai tesque) and subjected to ELISA. ELISA was performed according to the manufacture’s protocol (R&D SYSTEMS).

### qPCR analysis

Total RNA was extracted from isolated cells using the ReliaPrep RNA Miniprep System (Z6011; Promega, Madison, WI, USA) and reverse transcribed with SuperScript III (11752-250; Invitrogen, Thermo Fisher Scientific, Waltham, MA). qPCR was performed with a LightCycler (Roche, Basel, Switzerland) using SYBR Green (Toyobo, Osaka, Japan). The results were normalized to Gapdh expression level. The primers used were: *Gapdh*, 5′-TCCACCACCCTGTTGCTGTA-3′ and 5′-ACCACAGTCCATGCCATCAC-3′; *Tnfsf11*, 5′-AGCCATTTGCACACCTCAC-3′ and 5′-CGTGGTACCAAGAGGACAGAGT-3′.

### Analysis of the long bones

For micro-CT analysis, femurs were isolated from WT and RL-D4-KO mice and fixed in 70% ethanol. CT scanning was performed using the ScanXmate-A100S Scanner (Comscantechno). Three-dimensional microstructural image data were reconstructed, and structural indices were calculated using TRI/3D-BON software (RATOC). For histomorphometric analyses, tibias from WT and RL-D4-KO mice were dissected, fixed in 70% ethanol, and subjected to standard dynamic bone histomorphometric analyses as previously described.^58^ Toluidine blue and TRAP staining images were captured using the BZ-II Analyzer (Keyence Co., Osaka, Japan).

### ChIP-seq data and motif analyses

ChIP-seq data were downloaded from the GEO and ENCODE Project databases and visualized using the Integrative Genomics Viewer (version 2.3.12).^66^ Position weight matrices (PWMs) for motifs were obtained from the JASPAR database (https://jaspar.genereg.net/).^67^ We analyzed the transcription factor-binding sites within the input sequence of GRCh37 chr13: 43,058,344-43,062,075 using the JASPAR database. For motif identification, we employed FIMO^68^ with the default threshold value set to q < 10^−4^.

### Statistical analyses

Data were analyzed on GraphPad Prism software v9.5.1 and R software v4.3.1. Statistical tests, n-values, replicate experiments, and *P*-values are all indicated in the figures and/or legends. All data are expressed as the mean ± s.e.m. *P*-values were calculated using Student’s *t* test, analysis of variance (ANOVA) with Dunnett’s or Tukey’s multiple-comparison test (*P < 0.05; **P < 0.01; ***P < 0.001; ****P < 0.000 1, throughout the paper).

### Supplementary information

Supplementary Figure 1

Supplementary Figure 2

Supplementary Figure 3

Supplementary Figure legend

## Data Availability

All data that support the plots within this paper are available in the main text. The referenced publicly available datasets were downloaded from the Gene Expression Omnibus (GEO) (scRNA-seq data of human periodontal lesions; GSE164241, the epigenomic datasets of osteogenic cell epigenomic datasets; GSE51515, GSE54782, and GSM733779) and ENCODE Project databases (DNase-seq data of human PDL fibroblasts; ENCDO247AAA). The processed files of scRNA-seq data for mouse periodontitis are deposited in the GEO under accession GSE254766.
