# Supplementary figures and images for: The neutrophil–osteogenic cell axis promotes bone destruction in periodontitis
Source: Int J Oral Sci. 2024 Feb 27;16:18. doi: 10.1038/s41368-023-00275-8 (PMC10899642; doi:10.1038/s41368-023-00275-8)

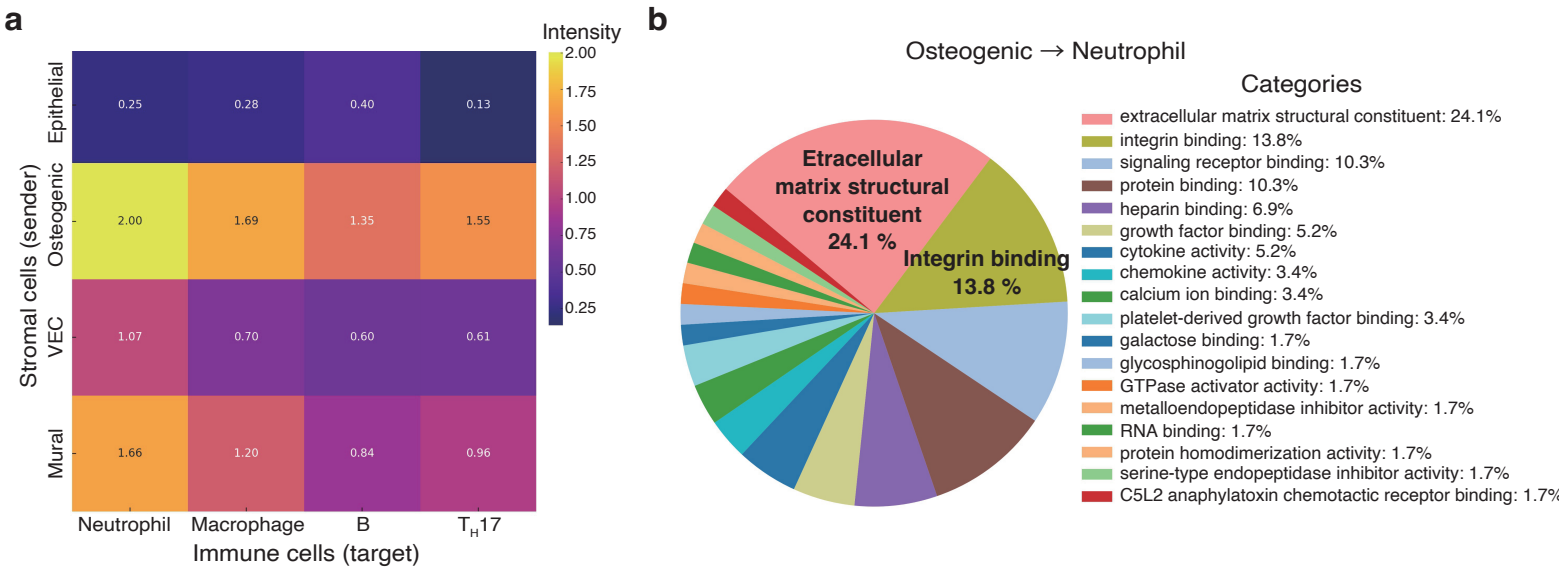

Supplementary Figure 1

Supplement: Supplementary file 1 — Supplementary Figure 1 [file 41368_2023_275_MOESM1_ESM.pdf]

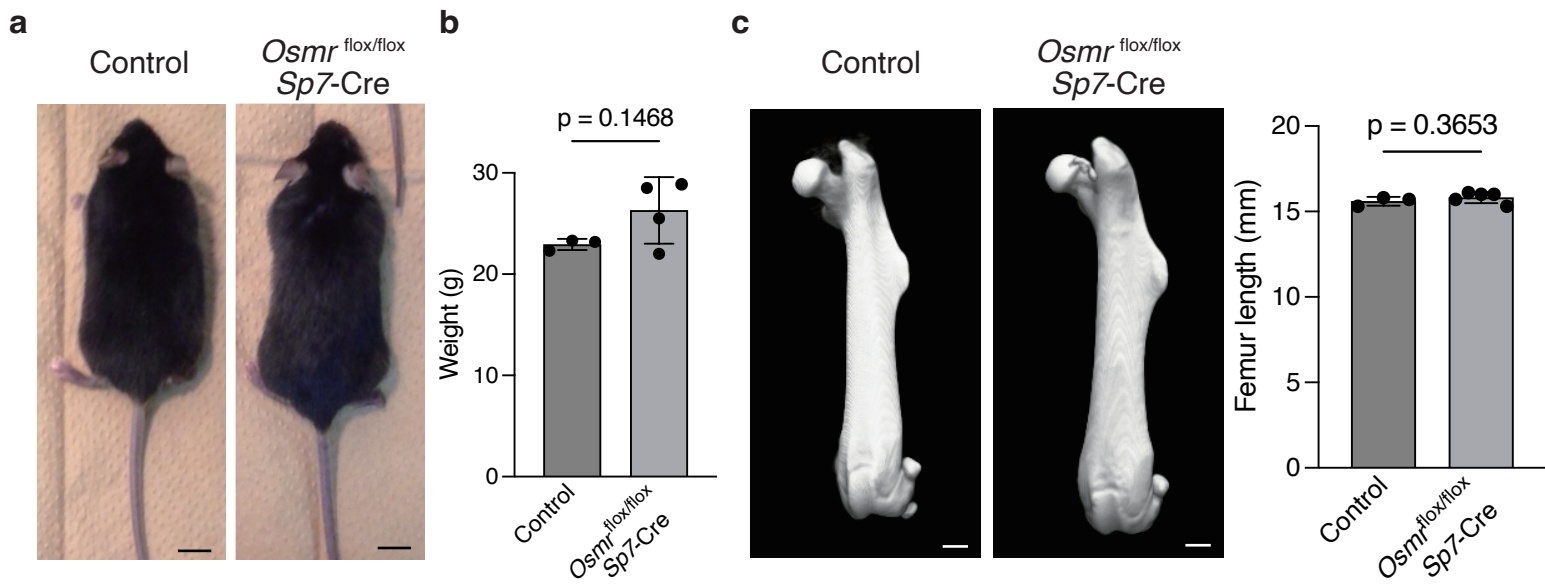

Supplement: Supplementary file 2 — Supplementary Figure 2 [file 41368_2023_275_MOESM2_ESM.pdf]
