# Supplementary material for: The neutrophil–osteogenic cell axis promotes bone destruction in periodontitis
Source: Int J Oral Sci. 2024 Feb 27;16:18. doi: 10.1038/s41368-023-00275-8 (PMC10899642; doi:10.1038/s41368-023-00275-8)
Supplement: Supplementary file 3 — Supplementary Figure 3 [file 41368_2023_275_MOESM3_ESM.pdf]

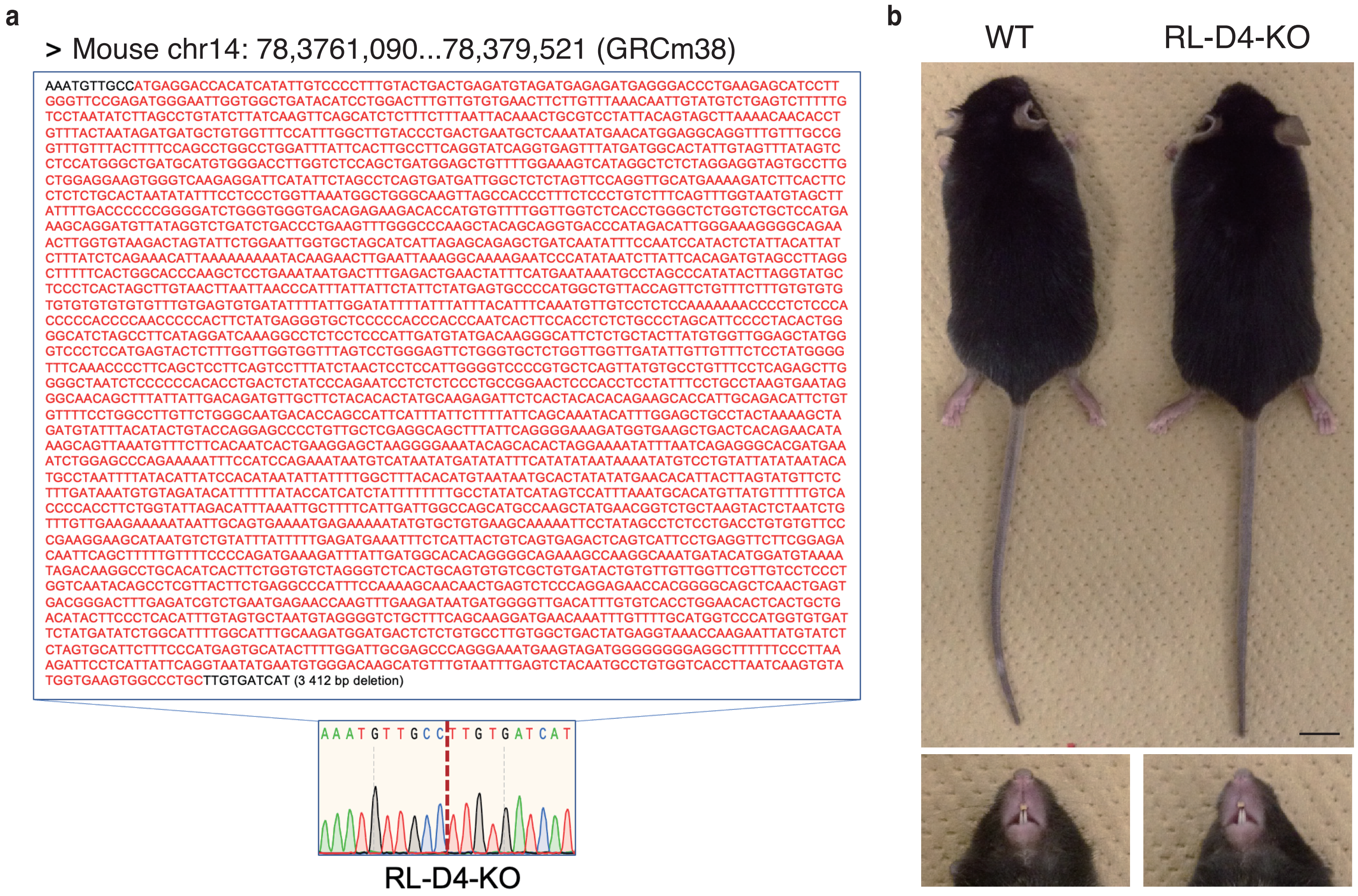

Supplementary Figure 3
