# Supplementary material for: The neutrophil–osteogenic cell axis promotes bone destruction in periodontitis
Source: Int J Oral Sci. 2024 Feb 27;16:18. doi: 10.1038/s41368-023-00275-8 (PMC10899642; doi:10.1038/s41368-023-00275-8)
Supplement: Supplementary file 4 — Supplementary Figure legend [file 41368_2023_275_MOESM4_ESM.docx]

**Figure S1. The effects of osteogenic cells on neutrophils in periodontitis. a,** Heat map showing the network intensity of interactions between stromal cells (sender) and immune cells (target). **b,** Proportion of Gene Ontology terms on molecules mediating the effect of osteogenic cells on neutrophils.

**Figure S2. The effects of inducible deletion of osteogenic cell *Osmr* on skeletal growth. a,** Representative macroscopic images of 10-week-old male control and *Osmr*^flox/flox^ *Sp7*-Cre mice. Scale bars, 1 cm. **b,** Body weight of 10-week-old male control and *Osmr*^flox/flox^ *Sp7*-Cre mice. **c,** Length of the femur of 10-week-old male control and *Osmr*^flox/flox^ *Sp7*-Cre mice. Scale bars, 1 mm.

**Figure S3. Generation of the RL-D4-KO mouse. a,** The nucleic acid sequence of RL-D4 in mice. The sequence region colored pink denotes the deletion region obtained with the CRISPR/Cas9 method. **b,** Representative macroscopic images of 6-week-old male WT and RL-D4-KO mice. Scale bars, 1cm
